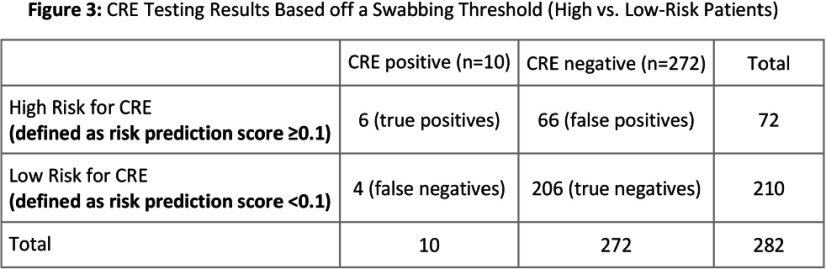# Validation of a Prediction Model to Identify Patients with Carbapenem-Resistant Enterobacterales upon Admission to Acute Care Hospitals

**DOI:** 10.1017/ash.2025.360

**Published:** 2025-09-24

**Authors:** Chris Bower, Hyun Bin Kim, Radhika Prakash Asrani, Twinkle Trehan, Chad Robichaux, Barney Chan, Boulis Madeleine, Sarah Satola, Jesse Jacob, Scott Fridkin, Jessica Howard-Anderson

**Affiliations:** 1 Emory University; 2 Emory University, School of Medicine; 1 Emory University; 3 Emory Healthcare and Emory University; 2 Emory University School of Medicine

## Abstract

**Background:** Rapid identification of patients with carbapenem-resistant Enterobacterales (CRE) on admission to an acute care hospital is critical to prompt initiation of infection control measures. Clinical risk prediction tools can assist in identifying high risk patients and allow facilities to perform targeted CRE screening. We aimed to prospectively validate a previously developed CRE risk prediction tool which incorporates data from current and prior hospital encounters and was incorporated into our electronic medical record (EMR). **Method:** From 6/2024 – 12/2024 we used an automated daily EMR report to calculate a CRE risk score (probability of a CRE clinical culture within 3 days of hospitalization) for all admissions from the previous day at two hospitals in an academic healthcare network in Atlanta, GA. On select days of the week, we approached a convenience sample of approximately 10 patients with the highest risk scores and obtained a peri-rectal swab on consented patients. Swabs were broth enriched and tested on CHROMagarTM ESBL plates. We used MALDI-TOF and/or the Vitek®2 GN74 panel for species identification and antibiotic susceptibility testing. To evaluate testing accuracy, we defined individuals as high-risk if they had a CRE risk prediction score in the top quartile of scores among patients approached. We calculated the sensitivity, specificity, and positive and negative predictive value of this threshold to predict patients with CRE peri-rectal carriage. **Results:** 9,422 admissions occurred on sampling days; we approached 720 of which 282 (39%) were consented and tested (Figure 1). Ten patients (3.5%) were positive for CRE: 4 Klebsiella pneumoniae, 3 Escherichia coli, 2 Enterobacter cloacae, and 1 Pantoea species. Among tested individuals, patients with CRE had a higher median CRE risk score (0.19% vs 0.04%), more healthcare exposures, a higher Elixhauser score, and more antibiotic days of therapy (Figure 2). Of the 72 (25%) patients at high-risk (CRE risk prediction ≥0.1%) 6 (8.3%) were CRE positive; using this threshold the sensitivity and specificity were 60% and 80%, respectively, and the positive and negative predictive value were 8% and 98%, respectively. **Conclusion:** Utilizing an EMR-based risk prediction tool can help identify patients at high-risk for CRE colonization. In healthcare facilities with a low CRE-prevalence, identifying a high-risk subset of patients, even with an 8% probability of CRE, could be a clinically meaningful infection prevention measure. Individual healthcare facilities could adjust the testing threshold based on the hospital and population needs.

**Figure:**
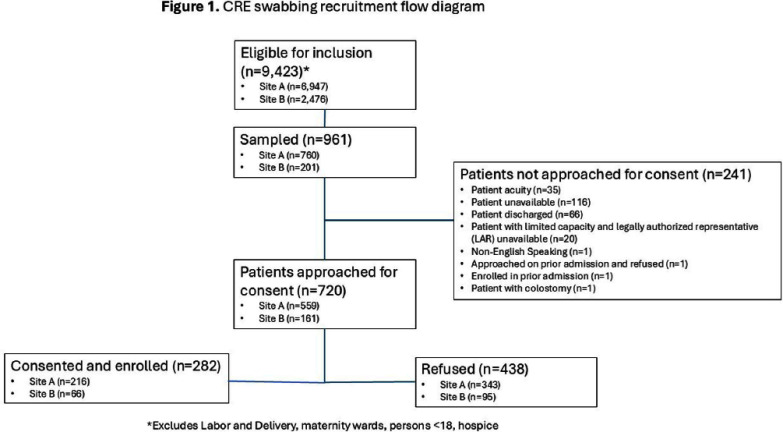

**Figure:**
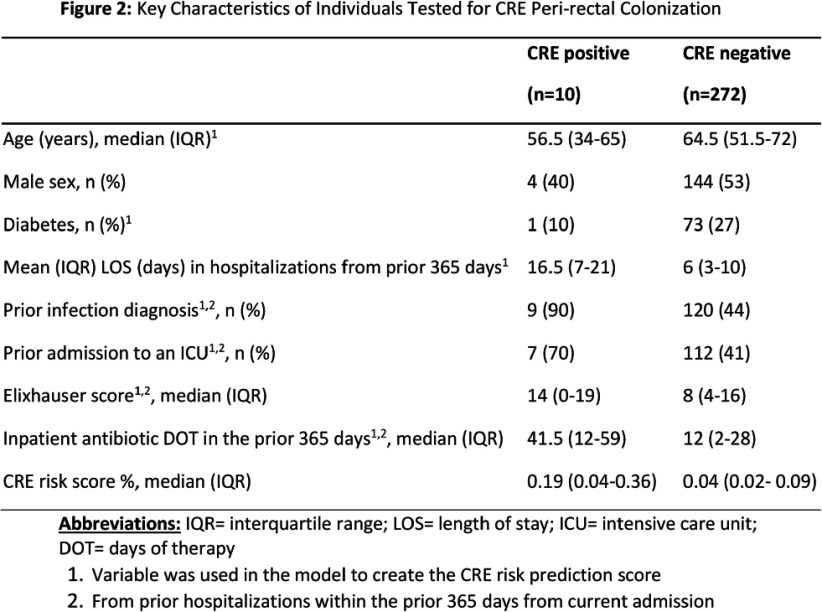

**Figure:**